# Supplementary material for: Capitella teleta gets left out: possible evolutionary shift causes loss of left tissues rather than increased neural tissue from dominant-negative BMPR1
Source: Neural Dev. 2024 May 2;19:4. doi: 10.1186/s13064-024-00181-7 (PMC11067212; doi:10.1186/s13064-024-00181-7)
Supplement: Supplementary file 2 — Supplementary Material 2. [file 13064_2024_181_MOESM2_ESM.pdf]

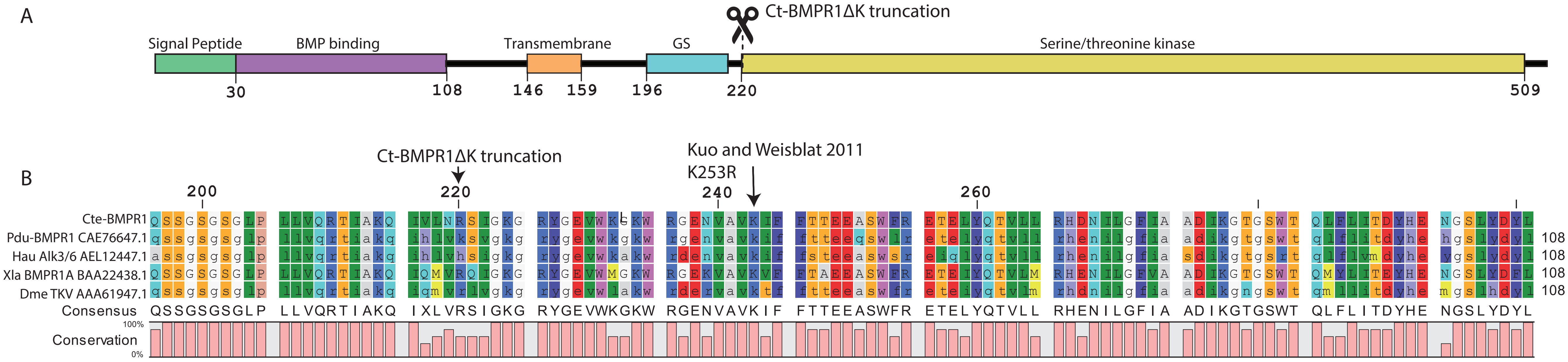

A
Ct-BMPR1ΔK truncation
Signal Peptide
BMP binding
Transmembrane
GS
Serine/threonine kinase
30
108
146
159
196
220
509
B
Ct-BMPR1ΔK truncation
Kuo and Weisblat 2011
K253R
200
220
240
260
Cte-BMPR1
Pdu-BMPR1 CAE76647.1
Hau Alk3/6 AEL12447.1
Xla BMPR1A BAA22438.1
Dme TKV AAA61947.1
108
108
108
108
Consensus
QSSGSGSGLP LLVQRTIAKQ IXLVRSIGKG RYGEVWKGKW RGENVAVKIF FTTEEASWFR ETELYQTVLL RHENILGFIA ADIKGTGSWT QLFLITDYHE NGSLYDYL
Conservation
100%
0%
