# Supplementary material for: Capitella teleta gets left out: possible evolutionary shift causes loss of left tissues rather than increased neural tissue from dominant-negative BMPR1
Source: Neural Dev. 2024 May 2;19:4. doi: 10.1186/s13064-024-00181-7 (PMC11067212; doi:10.1186/s13064-024-00181-7)
Supplement: Supplementary file 3 — Supplementary Material 3. [file 13064_2024_181_MOESM3_ESM.pdf]

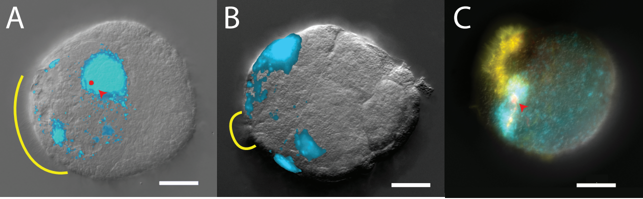

Supplementary Figure 2. Representative unelongated larvae after BMPR1ΔK-injection. A) Anterior view of animal with 1 eye pigment cell (red), and 1 presumed brain lobe. B) larva with no clear features or axes. C) Anterior view of animal with 1 eye sensory cell (red). Yellow: cilia; cyan: nuclei; red: larval eye; arrowheads: eyes; scale bars: 0.5 µm.
